# Metagenomic analysis of core differential microbes between traditional starter and Round-Koji-mechanical starter of Chi-flavor *Baijiu*

**DOI:** 10.3389/fmicb.2024.1390899

**Published:** 2024-06-17

**Authors:** Jinglong Liang, Lichuan Deng, Zhipu Li, Yongtao Fei, Weidong Bai, Wenhong Zhao, Songgui He, Rongbing Cao

**Affiliations:** ^1^Guangdong Provincial Key Laboratory of Lingnan Specialty Food Science and Technology, Zhongkai University of Agriculture and Engineering, Guangzhou, China; ^2^Key Laboratory of Green Processing and Intelligent Manufacturing of Lingnan Specialty Food, Ministry of Agriculture, Zhongkai University of Agriculture and Engineering, Guangzhou, China; ^3^Guangdong Jiujiang Distillery Co. Ltd., Foshan, China

**Keywords:** metagenomic, core differential microbes, traditional starter, Round-Koji-mechanical starter, Chi-flavor *baijiu*

## Abstract

*Xiaoqu* starter serves as the saccharifying and fermenting agent in the production of Cantonese soybean-flavor (Chi-flavor) *Baijiu*, and the complex microbial communities determine the flavor and quality of the product. Round-Koji-mechanical starter (produced by using an automated starter-making disk machine) is advantageous as it decreases operator influence, labor costs, and fermentation time, but the product quality is lower compared to traditional starter. Thus, two types of starters (traditional and Round-Koji-mechanical starter) from a Cantonese *Baijiu* factory were compared in a metagenomic analysis to investigate the differences in microbial community composition and core microbes. The results showed that several core microbes related to carbohydrate metabolism, amino acid metabolism and lipid metabolism, were differentially enriched in the traditional starter. *Mucor lusitanicus* and *Rhizopus delemar* were significantly positively correlated with the three key metabolic pathways. *Saccharomyces cerevisiae, Cyberlindnera fabianii, Kluyveromyces marxianus, Lactobacillus fermentum, Mucor ambiguous, Rhizopus microspores, Rhizopus azygosporus, Mucor circinelloides,* and *Ascoidea rubescens* were significantly positively correlated with two of the three key metabolic pathways. The results of this study provide a basis for understanding the differential core microbes in traditional and Round-Koji-mechanical starters of Chi-flavor *Baijiu*, and they also provide guidance for improving Round-Koji-mechanical starter.

## 1 Introduction

Chinese *Baijiu*, a long-standing alcoholic beverage, is considered one of the remarkable creations of ancient China (Xu et al., 2017). In the past five years, the total output of Chinese *Baijiu* has reached 37.85 million kiloliters. Chinese *Baijiu* is typically produced through solid-state fermentation using various grains as the initial ingredients. *Jiuqu* is a specific kind of starter used in the fermentation process of Chinese *Baijiu*. It is made up of various raw materials, microflora, enzymes, and aromatic precursor substances (Tu et al., 2022). The taste of Chinese *Baijiu* is derived from the existence of volatile and non-volatile compounds, primarily generated through microbial metabolism during the fermentation process. Volatile substances found in Chinese *Baijiu* include esters, alcohols, acids, aldehydes, nitrogen-containing compounds, sulfur-containing compounds, and terpenes. These compounds play a significant role in determining the aromatic characteristics and overall quality of Chinese *Baijiu*. Currently, over 2,400 chemicals have been identified in Chinese *Baijiu* that contribute to its flavor profile. Some of these chemicals, such as short-chain fatty acids, peptides, and phenols, have been found to have potential health benefits for humans (Sun et al., 2015; Fang et al., 2019; Xu et al., 2020; Jiang et al., 2021). The various types of Chinese *Baijiu* can be distinguished based on factors such as the production processes, raw materials, flavors, and region. According to the *Jiuqu* starter used, Chinese *Baijiu* can be classified as *Xiaoqu Baijiu*, *Daqu Baijiu*, *Maiqu Baijiu*, etc. (Zheng and Han, 2016). *Xiaoqu Baijiu* accounts for a sixth of Chinese *Baijiu*, and is mainly distributed in the southern region of China, including in Guizhou, Sichuan, and Hubei (Zhao et al., 2021). Rice, sorghum, and wheat are used in the production of *Xiaoqu Baijiu* (Su et al., 2010). These raw materials are cooked, mixed with microbes in a starter, and brewed by solid-state fermentation. *Xiaoqu* starter is the fermentation and saccharification agent for *Xiaoqu Baijiu* production. Compared to *Daqu* starter, it has fewer microbial species (mainly genera such as *Rhizopus*, *Trichoderma*, *Lactobacillus*, and yeast) (Gou et al., 2015) and a shorter fermentation cycle, and the flavors are not as rich (Xu et al., 2022).

Based on the production processes, raw materials, and other factors such as edaphoclimatic condition in different regions, there are many different types of *Xiaoqu Baijiu*, such as *Huaxia Xiaoqu*, *Hubei Xiaoqu*, *Dazhou Xiaoqu*, and *Jiujiang Xiaoqu* (Wu et al., 2017; Wang et al., 2018). The production of traditional *Xiaoqu* starter mainly involves manual processes, including steaming, spreading, and cooling the raw material (rice), adding crushed cake seeds, pressing the mixture into a disk shape, and incubating the product at 28–37°C for 3–5 days. As the traditional *Xiaoqu* starter production process is under non-asseptic conditions, the quality can vary greatly depending on the environment (Zheng and Han, 2016). In addition, the process entails high labor costs, involving many workers and high labor intensity.

With the progress of automated technology, the mechanical production of starter has gradually been developed, which includes automated mechanical disk-forming technologies and automated fermentation facilities, with the aim of reducing labor costs, standardizing the quality of starter, and achieving pollution-free production (Wang et al., 2018). However, the quality of *Jiujiang Xiaoqu* (Chi-flavor) *Baijiu* produced by Round-Koji-mechanical starter failed to reach the quality of *Baijiu* produced by traditional starter (Fei et al., 2023). Starter, which is a combination of many microbes and many enzymes (Shen, 1998; Yu, 2010), promotes simultaneous saccharification and fermentation during *Xiaoqu Baijiu* production. The quality and flavor composition of starter largely depend on the microbial community composition and metabolic functions (Jin et al., 2019). Exploring the differences in microbial community composition between mechanical and traditional starters, which can help for improving the quality of mechanical starters. An amplicon sequencing study compared the bacterial (but not fungal) diversity between traditional starter and the Round-Koji-mechanical starter of Chi-flavor *Baijiu*. The results showed that *Lactobacillus* and *Pediococcus* were dominant in both starters, *Weissella* was dominant in the traditional starter, and *Bacillus*, *Acetobacter*, *Acinetobacter*, and *Klebsiella* were dominant in the mechanical starter (Wang et al., 2018). Additionally, our team compared the bacterial diversity in the prophase of Chi-flavor *Baijiu* fermentation between the two starters, and we found that *Lactobacillus* and *Saccharomyces* were the dominant genera in both starters, but *Pediococcus* and *Weissella* were enriched in the traditional starter compared to the mechanical starter (Fei et al., 2023). However, the core microbial species in traditional starters remain unknown.

Amplicon sequencing involves using PCR technology to amplify the target in the samples. It is often used to analyze the 16S rRNA and ITS sequences of bacterial or fungal communities, but it is only accurate to the genus level (Caporaso et al., 2011). Metagenomic analysis based on amplicon or high-throughput sequencing involves collecting genome-wide data from diverse organisms in a given sample (Walsh et al., 2017). Metagenomic analysis based on high-throughput sequencing can achieve species-level analysis, and various analytical methods can be used to process and analyze metagenomic data for targeted microbial research (Franzosa et al., 2015).

A comparative analysis of physical factors in our team showed that the saccharifying, esterification, and fermentation capacities were significantly higher in traditional starter than Round-Koji-mechanical starter (Wang et al., 2023). In the current study, the differences in microbial community composition between traditional and mechanical starters were analyzed by metagenomic analysis. In addition, the core differential genes related to carbohydrate, lipid, and amino acid metabolism (based on the KEGG database) were identified, and a correlation analysis of the core differential genes was conducted using the NR database, which was mined to determine the core differential microbes between traditional and mechanical starters. The core advantageous differential microbes between the traditional and mechanical starters were identified, and the results provide a basis for improving the quality of Round-Koji-mechanical starter and promoting the industrialization and mechanization of Chi-flavor *Baijiu* production.

## 2 Materials and methods

### 2.1 Sample collection

Two types of starters contained traditional starter (BQ) and mechanical starter (Round-Koji-mechanical starter, SQ), were collected from *Jiujiang* Distillery in Foshan City, Guangdong, China. Regarding BQ, a batch with a starter was used for three-point sampling (for example, three pieces from the top, middle and bottom of the starter). Regarding SQ, a batch with a starter was used for five-point random sampling.

### 2.2 Genomic DNA extraction

The collected samples were sent to Shanghai Meiji Biotechnology Company (Shanghai City, China) for DNA extraction. Briefly, each sample was weighed at −80°C to obtain 10 g. DNA was extracted using an E.Z.N.A.^®^ Soil DNA Kit according to the manufacturer’s instructions. DNA quality was measured by a microspectrophotometer (NanoDrop2000, Thermo Fisher Scientific) and 1% agarose gel electrophoresis.

### 2.3 High-throughput sequencing, quality control, and assembly

The extracted DNA was sent to Shanghai Meiji Biological Company for sequencing and data processing. Briefly, raw metagenomic data were obtained using an Illumina HiSeq 4000 sequencing system. The raw data were subjected to quality control using Fast Software Version 0.12.0 (Babraham Institute, UK), which removed the adapter sequences from the 3′ and 5′ ends and the reads that were < 50 bp, had a mean quality <20, or contained N bases, retaining the high-quality paired- and single-end reads. To obtain high-quality clean data, using BWA software (Li and Durbin, 2009), the reads were compared to the DNA sequences of the raw brewery materials, and reads with high similarity (contaminants) were removed. Contigs were obtained using the Multiple MEGAHIT splicing strategy, and the contigs ≥300 bp were selected as the final assembly results. Open reading frame (ORF) prediction of the contigs was performed using Prodigal software (Hyatt et al., 2010). Next, clustering was performed using CD-HIT software, and the longest gene in each cluster was selected as the representative sequence to construct a non-redundant gene set. Finally, the high-quality reads in each sample were compared to the non-redundant gene set (default criterion: 95% identity) using SOAPaligner/soap2 Version 2.21 (Beijing Genomics Institute, China).

### 2.4 Taxonomic assignment

The DIAMOND software (Buchfink et al., 2015) was used to compare the non-redundant gene set to the NR database,^1^ and species annotations were obtained from the corresponding Taxonomy database of the NR database. Based on this information, relative abundances at the domain, kingdom, phylum, class, order, family, genus, and species levels in each sample were calculated. Next, the abundance of the species in each sample was counted at each taxonomic level to construct an abundance table at the corresponding taxonomic level and complete species annotation.

### 2.5 Kyoto encyclopedia of genes and genomes (KEGG) functional annotation

The KEGG database^2^ was used to identify sets of genes related to the three major metabolic pathways, i.e., carbohydrate, lipid, and amino acid metabolism. Next, for gene annotation, DIAMOND was used to compare the abovementioned non-redundant gene set to the KEGG database, including the carbohydrate, lipid, and amino acid metabolism-related genes.

### 2.6 Identification of core differential microbes based on core differential genes

The functional composition and clustering of the starter samples, based on genes related to carbohydrate, lipid, and amino acid metabolism, were analyzed by Circos Analysis, Clustering Analysis, and Principal Component Analysis (PCA). Functional genes unique to either starter were identified by constructing Venn Diagrams and by conducting significant difference tests. Linear Discriminant Analysis (LDA) was used to analyze the effect sizes. Based on these analyses, core differential genes related to carbohydrate metabolism, amino acid metabolism and lipid metabolism, were identified. Correlation analysis was conducted using the data on these core differential genes and the NR database. The Network Complex Analysis Toolkits (Python package) were used to calculate the correlations between the genes and microbial species. Consequently, the differential core microbial species in traditional starter (BQ) were determined based on the strength of the correlations.

## 3 Results and discussion

### 3.1 Overview of metagenomic data

After sequencing the two starters (Supplementary Table S1), there were 263,860,016 raw reads (39,842,862,416 bp) and after quality control, there were 2,586,116,640 clean reads (38,934,390,577 bp). There were 941,901 contigs >300 bp (1,080,460,208 bp). The mean N50 and N90 were 2,152 and 425 bp, respectively. 1,462,862 ORFs were predicted, with a total length of 641,017,419 bp. The distribution of non-redundant gene lengths in Supplementary Figure S1 indicates that the number of sequences decreased with gene length.

### 3.2 Taxonomic analysis

A total of 3 domains, 5 kingdoms, 16 phyla, 30 classes, 46 orders, 83 families, 134 genera, and 321 species were annotated by NR species annotation of the two starters. The 3 domains were Bacteria, Eukarya and Viruses.

In BQ, the relative abundance of fungi accounted for 72% and that of bacteria accounted for 28%. In SQ, both accounted for about 50%. Environmental factors such as temperature and humidity changed more slowly in SQ than BQ. It was hypothesized that the reason for the different community composition between the two starters was the environment during fermentation. These results regarding relative abundance of domains is consistent with the results of Hu Y. L. et al. (2021).

The results of phylum level were showed in Supplementary Figure S2A. There are 10 phylas with relative abundance greater than 0.1%. The relative abundance of *Mucoromycota* accounted for 65.4% in BQ and that for 46.4% in SQ. The relative abundance of *Firmicutes* accounted for 25.8% in BQ and that for 46.7% in SQ. Thus, the *Mucoromycota* and *Firmicutes* were dominant in both starters. In a previous studies of *Xiaoqu* (Su et al., 2010; Wu et al., 2017), the dominant phyla were *Firmicutes, Actinobacteria, Proteobacteria, Ascomycota, Mucoromycota, Basidiomycota*, which is similarity with the results of our study.

The results of genus level were showed in Supplementary Figure S2B. There are 10 genus with relative abundance greater than 0.1%. The relative abundance of *Rhizopus* accounted for 50.3% in BQ and that for 31.1% in SQ. The relative abundance of *Lactobacillus* accounted for 22.5% in BQ and that for 18.3% in SQ. The relative abundance of *Mucor* accounted for 8.5% in BQ and that for 4.9% in SQ. Thus, the *Rhizopus*, *Lactobacillus,* and *Mucor* were dominant in both starters. However, the abundance of *Bacillus* accounted for 21.1% and only showed in SQ. The mechanical process uses a large blower to control humidity, a large amount of air can be blown into the starter. This condition was good for growthing of aerobic microorganisms, such as *Bacillus*. These results were consistent with the results of a previous study about mechanical Daqu (Zuo et al., 2020).

**Figure 1:**
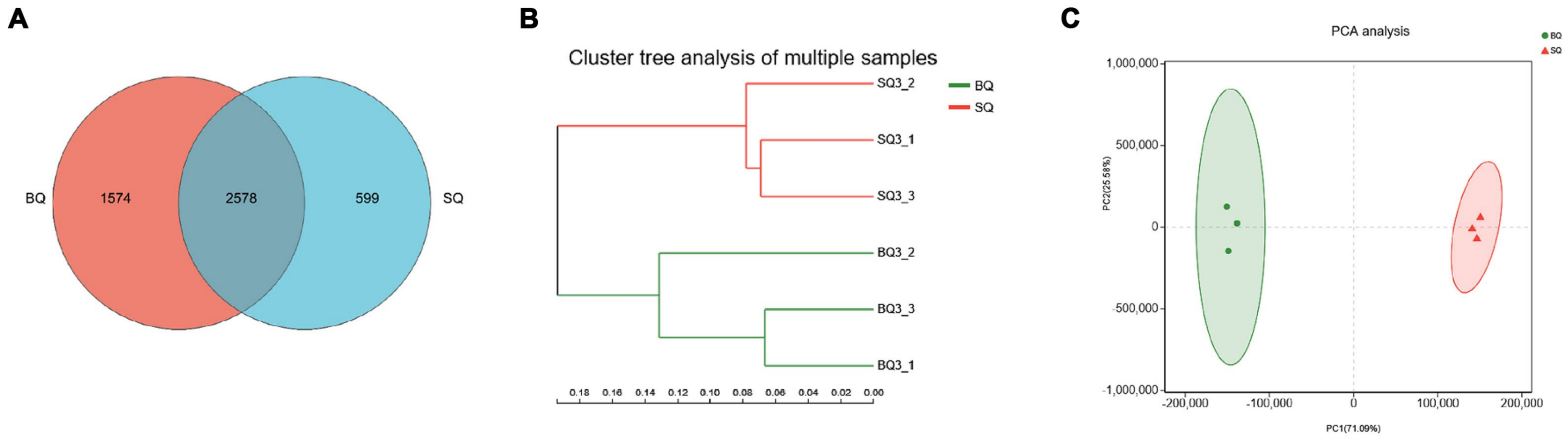
Analyses of microbial communities at the species level in the two starters. **(A)** Venn diagram, **(B)** PCA clustering results, and **(C)** PCA score plot.

Metagenomic sequencing technology allowed species-level analyses of the two starters. According to the Venn diagram, there were 1,574 unique species in BQ, 599 unique species in SQ, and 2,578 common species (Figure 1A). According to the PCA (Figures 1B,C), the confidence intervals were far apart, indicating that the two starters were significantly different.

**Figure 2:**
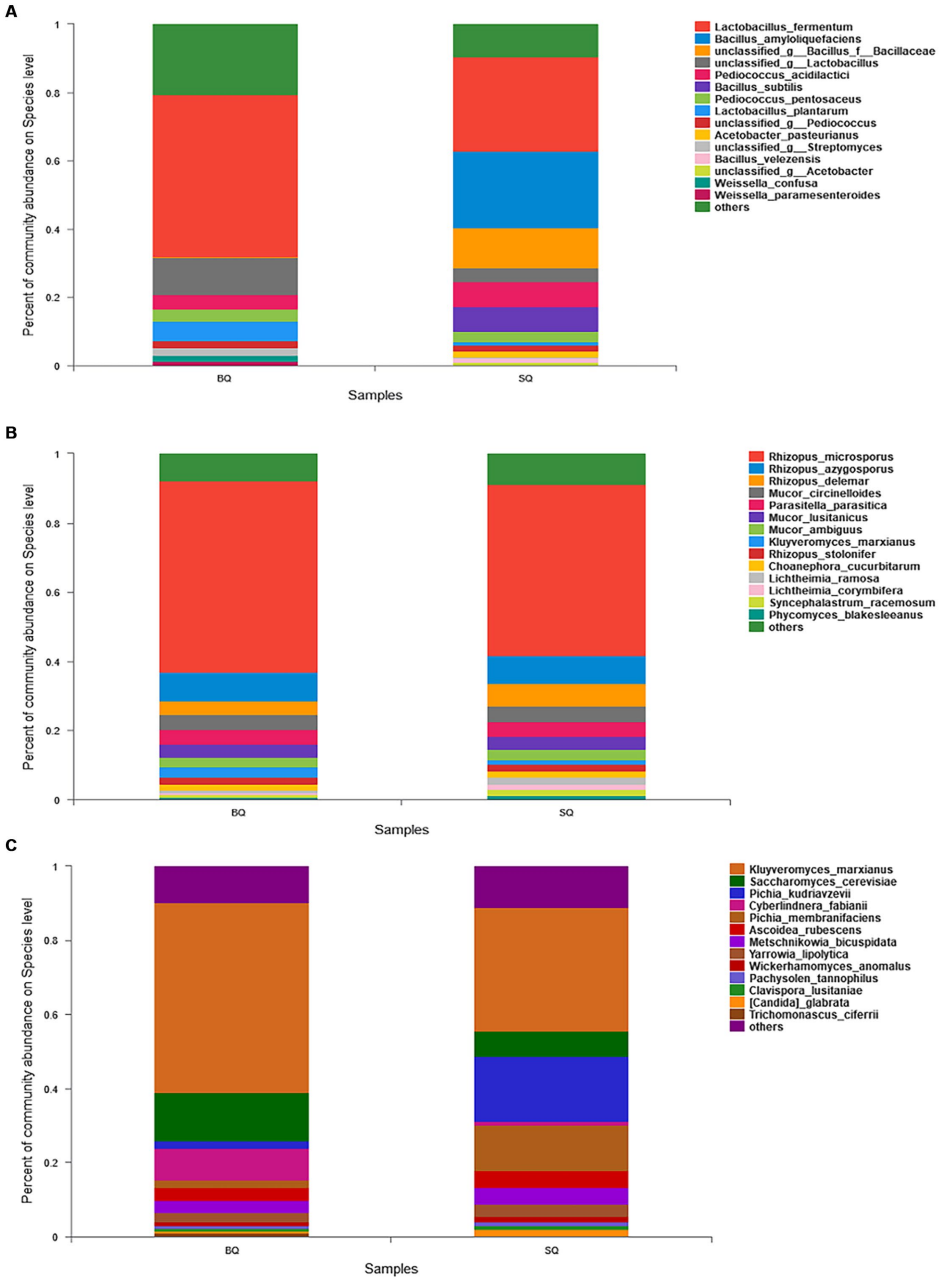
Relative abundances of **(A)** bacteria, **(B)** molds, and **(C)** yeast at the species level between the two starters.

In the species composition analysis of bacteria (Figure 2A), the abundance of *Lactobacillus fermentum* was higher in BQ (47.3%) than SQ (27.8%). This species is associated with volatile flavors such as 3-methyl-1-butanol, 2-methyl-1-propanol, and phenylethanol in yellow *Baijiu* (Wenhong, 2018). The abundance of *Lactobacillus plantarum* was also higher in BQ (5.8%) than SQ (1.1%). This species can produce certain flavor precursors and can survive at low pH and high alcohol concentrations in *Baijiu* fermentation (Todorov and Franco, 2010). Moreover, *Weissella confusa* and *Weissella paramesenteroides* were not present in SQ, whereas *Bacillus amyloliquefaciens*, *Bacillus subtills, Acetobacter Bacillus amyloliquefaciens, Bacillus subtills, Acetobacter pasteurianus,* and *Bacillus velezensis* were not present in BQ. The dominant species in SQ were *Bacillus amyloliquefaciens* and *Bacillus subtills*. These two species can produce α-amylase and glucoamylase for raw material saccharification and hydrolysis (Li et al., 2014). *Bacillus subtills* and *Bacillus velezensis* inoculation of macroalgae (for biofortification) enhanced saccharification, ethanol fermentation, and aroma formation (He et al., 2019). However, massive reproduction of *Bacillus* in starter inhibiting growth of *Lactobacillus*, *Pediococcus* and *Weissella* (Wang et al., 2015).

In the species composition analysis of molds (Figure 2B), there was no significant difference between the two starters. Yeast is important in *Baijiu* fermentation, so a species composition analysis of yeast (Figure 2C) was conducted. The abundance of *Kluyveromyces marxianus* was higher in BQ (51%) than SQ (32%). This species can produce many enzymes such as *β*-galactosidase, lipase, protein phosphatases, carboxypeptidase, and *β*-glucosidase (Karim et al., 2020). Therefore, it can catalyze the conversion of large molecules into small molecules and the bioproduction of flavor compounds. In addition, the abundance of *Saccharomyces cerevisiae* was also higher in BQ (18%) than SQ (10%). This species can produce ethanol and esters in fermented foods (Annan et al., 2003).

### 3.3 Distribution of genes associated with KEGG pathways in the two starters

Annotating the metagenomic data with KEGG level 1 pathways showed that the metabolism pathway had the highest abundance of functional genes, while the environmental processing pathway had the lowest (Supplementary Figure S3A). Annotating the metagenomic data with KEGG level 2 metabolism pathways showed that there was a high abundance of functional genes related to carbohydrate metabolism, amino acid metabolism, energy metabolism, lipid metabolism, metabolism of other amino acids, metabolism of cofactors and vitamins, and so on (Supplementary Figure S3B). Among these pathways, carbohydrate metabolism maintains microbial viability during fermentation, providing important compounds for cell structure, providing energy, producing ethanol, etc. Therefore, it is quite reasonable that the abundance of functional genes is highest in this pathway. These results regarding high abundance of functional genes related to carbohydrate metabolism were similar to previous results concerning *Guizhou Xiaoqu* (Liu et al., 2019) and *Dazhou Xiaoqu* (Xie et al., 2020). Amino acids are important for production of specific flavors, and have a large impact on brewery quality. Cofactors provide redox carriers for biosynthesis and catabolism and play an important role in energy transfer in microbial cells (Wang et al., 2013). Vitamins are often involved in metabolic processes in the form of cofactors. Therefore, there was a high abundance of functional genes related to cofactor and vitamin metabolism.

Regarding the KEGG level 1 pathway annotation, some genes were found to be associated with human diseases. Similar results have been found in other studies of various fermented foods, such as Korean Rice-flavor *Baijiu* (Kim et al., 2015), fermented sweet wort (Menz et al., 2010), and traditional fermented foods from northeastern India (Keisam et al., 2019). However, the presence of these genes does not imply that these foods are pathogenic to humans (Olano et al., 2001). In the case of yellow *Baijiu* fermentation (Liu et al., 2019), the genes associated with human diseases increased and then decreased with fermentation time, which may imply that the microbes or raw materials with genes associated with human diseases are affected by ethanol, *Streptococcus*, yeasts, *Saccharopolyspora, Aspergillus*, and other environmental factors (Vara and Hutchinson, 1988; Flewelling et al., 2015; Phongphakdee and Nitisinprasert, 2015). In addition, a wide variety of starter and *Baijiu* have been used safely for more than 9,000 years (McGovern et al., 2004), so the safety of *Xiaoqu* starter can be guaranteed to a certain extent.

### 3.4 Differential functional genes between the two starters

Genes related to carbohydrate, amino acid, and lipid metabolism were annotated using the KEGG database.

**Figure 3:**
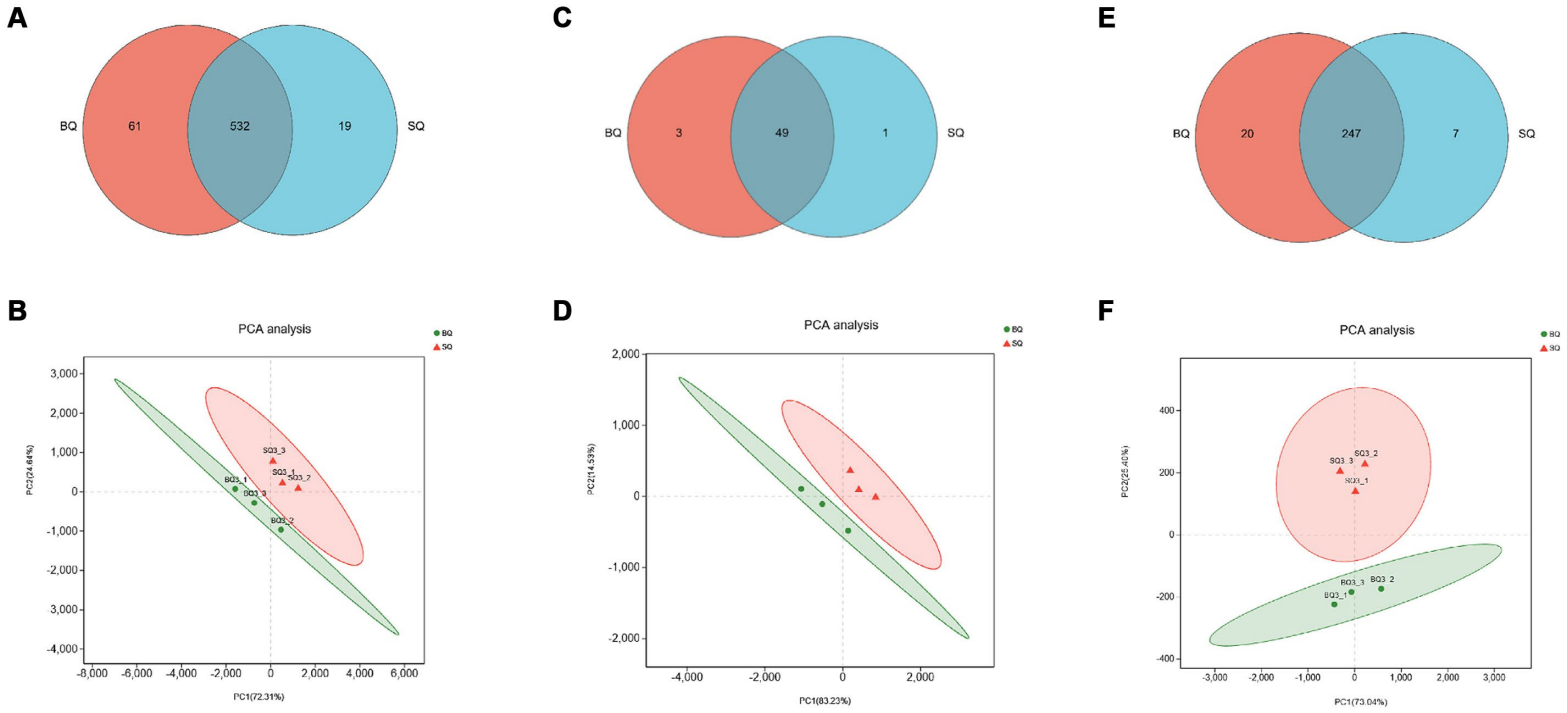
Analyses of genes related to three key metabolic pathways in the two starters. Venn diagrams of genes related to **(A)** carbohydrate, **(C)** amino acid, and **(E)** lipid metabolism. PCA score plots of starter samples based on genes related to **(B)** carbohydrate, **(D)** amino acid, and **(F)** lipid metabolism.

According to the Venn diagram of genes related to carbohydrate metabolism, there were 61 unique genes in BQ, 19 unique genes in SQ, and 532 common genes (Figure 3A). The PCA confidence intervals were far apart, indicating that the two starters were significantly different (Figure 3B). The LDA identified differential carbohydrate metabolism-related genes. Among the genes that were unique to BQ, >2% were functional genes, including 4-olyl-4-methyl-2-oxoglutarate aldolase (K10218), 2-dehydro-3-deoxygalactose phosphokinase (K00883), D-alpha-alpha-alpha-alpha-6-phosphate differential isomerase (K017195), butanol dehydrogenase (K00100), N-acetylglucosaminoglucosan kinase (K00884), 1,3-propanol dehydrogenase (K00086), succinate dehydrogenase (K00246), α-1-phospho-maltose synthase (K16148), starch synthase (maltosyltransferase) (K16147), and alditol oxidase (K00594). In BQ, the one gene with LDA ≥4 was chitin synthase (K00698), the genes with LDA ≥3 were β-glucosidase (K05349), 1,3-β-glucan synthase (K00706), galactosidase (K07407), malate dehydrogenase (K00029), glycogen synthase (K00693), and 1,3-β-glucosidase (K01210), and the genes with LDA ≥2.5 were glycosylase (K01178), malate dehydrogenase (K00026), glycogen debranching enzyme (K01196), fructose-6-phosphate-2-kinase (K19029), isocitrate decomposing enzyme (K01637), isocitrate dehydrogenase (K00031), malate synthase (K01638), pectinase (K01184), pyruvate kinase (K00873), β-phosphoglucan translocase (K01838), succinate dehydrogenase (K00234), acetyl coenzyme A hydrolase (K01067), (K00830), succinate CoA synthase (K01900), lactate dehydrogenase (K00101), pyruvate dehydrogenase (K21618), ethanol dehydrogenase (K00001), glycogenin (K00750), and 6-phosphofructo-2-kinase (K00900). The set of differential carbohydrate metabolism-related genes in BQ were related to pathways such as the glycolysis pathway, citric acid cycle pathway, glyoxylate pathway, starch synthesis and catabolism pathway, fructose and maltose metabolism pathway, and lactose metabolism pathway. Cellulose, starch, dextrin, maltose, sucrose and other carbon source substances of raw materials can be metabolized into glucose or enter the glycolytic pathway. Therefore, it can be inferred that BQ has a better ability to utilize carbon sources than SQ, with higher levels of saccharification, liquefaction and fermentation. This is consistent with our previous research (Wang et al., 2023).

According to the Venn diagram of genes related to amino acid metabolism, there were 3 unique genes in BQ, 1 unique gene in SQ, and 49 common genes (Figure 3C). The PCA confidence intervals were far apart, indicating that the two starters were significantly different (Figure 3D). The LDA identified differential amino acid metabolism-related genes, including asparaginase (K13051), 2,4-diaminovaleric acid dehydrogenase (K21672), 3-hydroxyacyl-coenzyme A dehydrogenase (K01825), 3-hydroxyisobutyl-CoA hydrolase (K05605), glutamate decarboxylase (K01580), primary amine oxidase (K00277) and glutamine oxidase (K00279), oxidase (K00276), cytoplasmic aminopeptidase (K11142), 4-aminobutyric acid aminotransferase (K13524), glutamate cysteine ligase catalytic subunit (K11204), and cystathionine gamma-cleaving enzyme (K01758). The set of differential amino acid metabolism-related genes in BQ were related to the generation of proline, glutamic acid, cysteine, and glutathione. Amino acids undergo Maillard reaction with reducing sugars, producing various compounds such as furans, pyrans, pyrazines, aldehydes, ketones, etc., which can increase the content of flavor substances in *Baijiu* (Huang et al., 2024). In addition, relevant reports have found that glutathione can reduce the loss of esters and terpenes in *Baijiu*, thereby increasing the content of aroma components (Webber et al., 2014).

According to the Venn diagram of genes related to lipid metabolism, there were 20 unique genes in BQ, 7 unique genes in SQ, and 247 common genes (Figure 3E). The PCA confidence intervals were far apart, indicating that the two starters were significantly different (Figure 3F). The LDA identified differential lipid metabolism-related genes, including fatty acid synthase (K11533), acyl coenzyme A dehydrogenase (K06445), stearoyl coenzyme A desaturase (K22770), 3-phosphoglycerol dehydrogenase (K00112, K00113), glycerol kinase (K00864), acyl coenzyme A oxidase (K00234), cysteine gamma-cleaving enzyme (K01758), acyl coenzyme A oxidase (K00232), fatty acid synthase (K00667), acetyl CoA carboxylase (K11262), and acyl coenzyme A dehydrogenase (K00249). The set of differential lipid metabolism-related genes in BQ were related to pathways such as the fatty acid biosynthesis pathway and fatty acid oxidation pathway. Fatty acids and acetyl CoA can serve as precursors for ester synthesis. Therefore, it can be inferred that BQ has a better esterification ability than SQ. This is consistent with our previous research (Fei et al., 2023; Wang et al., 2023).

**Figure 4:**
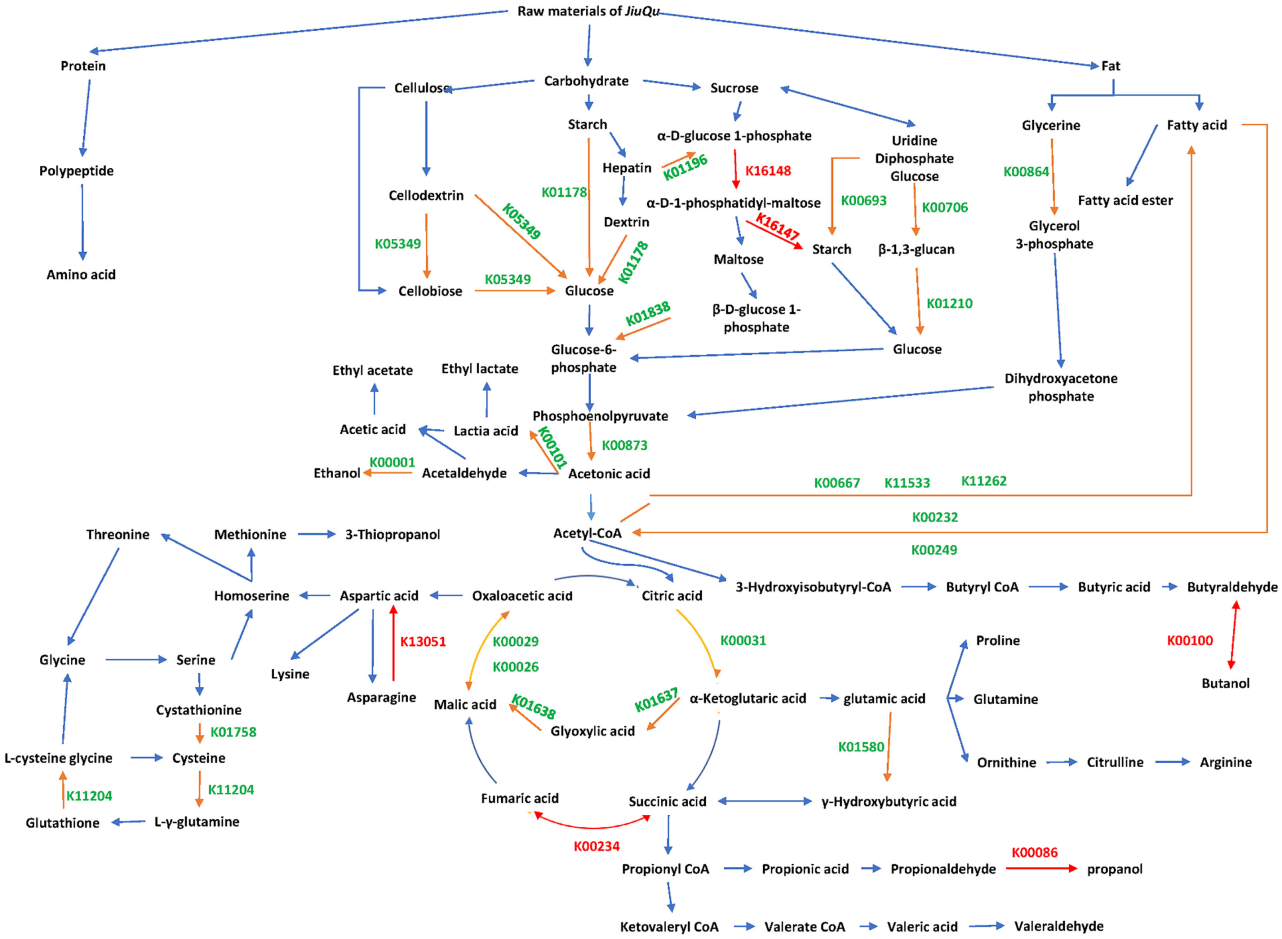
Metabolic network of differential genes between the two starters. Orange, green, and red KEGG orthology entries indicate dominant differential functional genes, dominant differential genes, and unique functional genes, respectively.

A metabolic network of these differential genes is shown in Figure 4. There are a total of 24 dominant differential functional genes, 28 dominant differential genes, and 6 unique functional genes in BQ, respectively.

### 3.5 Identifying core differential microbes based on core differential genes

The core differential microbes related to carbohydrate metabolism between the two starters are shown in Table 1. Correlation analysis of carbohydrate metabolism-related differential genes, using the NR database, was performed using a *p*-value of 0.05 (Supplementary Figure S4). Regarding carbohydrate utilization, there were 4 yeast species (*Saccharomyces cerevisiae, Cyberlindnera fabianii, Kluyveromyces dobzhanskii,* and *Kluyveromyces marxianus*), 4 bacterial species (*Puccinia cinerea CS2, Microbacterium Indicum, Lactobacillus fermentum,* and *Lactobacillus mucilaginosus*), and 4 mold species (*Mucor lusitanicus* and *Mucor ambiguus, Rhizopus microsporus,* and *Rhizopus azygosporus*). Of these, *Saccharomyces cerevisiae, Cyberlindnera fabinaii, Rhizopus microsporus, Rhizopus azygosporus,* and *Mucor lusitanicus* were positively correlated with genes of multiple enzymes. *Saccharomyces cerevisiae, Cyberlindnera fabinaii* and *Rhizopus microsporus* were shown to be the core microbes for saccharification and liquefaction of starter, as they were associated with genes of K00693, K00706 and K01196. Regarding glycolysis, citric acid cycle, and glyoxylate cycle, *Ascoidea rubescens*, *Lichtheimia ramosa*, *Rhizopus azygosporus*, *Rhizopus microsporus*, *Trichomonascus ciferrii*, *Kluyveromyces marxianus*, *Lactobacillus fermentum*, *Mucor ambiguus*, *Diutina rugosa*, *Rhizopus delemar*, *Mucor circinelloides* were positively correlated with functional genes of these metabolism. *Rhizopus microsporus, Kluyveromyces marxianus, Lactobacillus fermentum and Mucor ambiguus* were consistent with the core microbes discovered in carbohydrate utilization, which indicating that these microorganisms may play a key role in carbohydrate metabolism in BQ. However, there were fewer positively correlated microbes in energy metabolism than in the carbohydrate utilization. Although energy metabolism is necessary for all microbes, some of the microbes were negatively correlated, which may be related to mutual antagonism among the microbes.

**Table 1:** Core differential microbes related to carbohydrate metabolism in BQ.

KO	Name	Core microbes
K00693	Glycogen synthase	*Saccharomyces cerevisiae* *Cyberlindnera fabianii* *Mucor lusitanicus* *Mucor ambiguous*
K00706	1,3-β-Glucan synthase	*Cyberlindnera fabianii* *Saccharomyces cerevisiae* *Rhizopus microsporus*
K01196	Glycogen debranching enzyme	*Cyberlindnera fabianii* *Mucor lusitanicus* *Saccharomyces cerevisiae* *Kluyveromyces dobzhanskii*
K01210	1,3-β-Glucosidase	*Rhizopus azygosporus* *Rhizopus microsporus* *Lactobacillus mucosae*
K01178	Glycosylase	*Saccharomyces cerevisiae* *Cyberlindnera fabianii*
K01838	β-Phosphoglucan translocase	*Lactobacillus fermentum*
K05349	β-Glucosidase	*Rhizopus microsporus* *Rhizopus azygosporus* *Lactobacillus mucosae* *Kluyveromyces marxianus*
K16147	starch synthase (maltosyltransferase)	*Microbacterium Indicum*
K16148	α-1-Phospho-maltose synthase	*Brevibacterium* sp. *CS2*
K00029 K00026	Malate dehydrogenase	*Rhizopus microsporus* *Mucor ambiguous* *Rhizopus delemar* *Mucor circinelloides*
K01637	Isocitrate decomposing enzyme	*Diutina rugosa*
K00031	Isocitrate dehydrogenase	*Lactobacillus fermentum* *Kluyveromyces marxianus*
K01638	Malate synthase	*Trichomonascus ciferrii*
K00873	Pyruvate kinase	*Lichtheimia ramosa*
K00234	Succinate dehydrogenase	*Rhizopus azygosporus* *Rhizopus microsporus*
K01900	Succinate CoA synthase	*Ascoidea rubescens*

The core differential microbes related to amino acid metabolism between the two starters are shown in Table 2. As the variability of amino acid metabolism was lower than that of carbohydrate metabolism, the *p* value for the correlation analysis was increased to 0.5 (Supplementary Figure S5). The core differential microbes related to amino acid metabolism were 6 mold species (such as *Rhizopus microsporus*, *Rhizopus azygosporus*, *Rhizopus delemar*, *Actinomucor elegans*, *Mucor lusitanicus,* and *Lichtheimia ramosa*). *Rhizopus microsporus*, *Rhizopus azygosporus*, *Rhizopus delemar* were consistent with the core differential microbes related to carbohydrate metabolism, suggesting that these microbes play a key role in BQ. *Mucor lusitanicus,* and *Lichtheimia ramosa* were consistent with the core differential microbes related to energy metabolism in the carbohydrate correlation analysis, so these microbes were also important. In addition, *Lactobacillus fermentum, Saccharomyces cerevisiae*, and *Kluyveromyces marxianus* were the core differential microbes related to carbohydrate metabolism, so these microbes may play a key role in BQ.

**Table 2:** Core differential microbes related to amino acid metabolism in BQ.

KO	Name	Core microbes
K13051	Asparaginase	*Serratia rubidaea*
K05605	3-Hydroxyisobutyl-CoA hydrolase	*Rhizopus microsporus*
K01580	Glutamate decarboxylase	*Rhizopus azygosporus* *Lactobacillus fermentum* *Saccharomyces cerevisiae* *Rhizopus microsporus*
K11142	Cytoplasmic aminopeptidase	*Rhizopus azygosporus* *Rhizopus microsporus* *Rhizopus delemar* *Actinomucor elegans* *Mucor lusitanicus*
K01758	Cystathionine γ-cleaving enzyme	*Rhizopus microsporus* *Unclassified Streptomyces* *Kluyveromyces marxianus*
K11204	Glutamate cysteine ligase catalytic subunit	*Rhizopus microsporus* *Lichtheimia ramosa*

The core differential microbes related to lipid metabolism between the two starters are shown in Table 3. As the variability of lipid metabolism was lower than that of carbohydrate metabolism, the p value for the correlation analysis was increased to 0.1 (Supplementary Figure S6). The core differential yeast species related to lipid metabolism were *Lachancea kluyveri* and *Cyberlindnera fabianii*, *Cyberlindnera fabianii* was also related to carbohydrate metabolism. The core differential mold species related to lipid metabolism were *Mucor ambiguous*, *Ascoidea rubescens*, *Mucor lusitanicus*, *Rhizopus stolonifer*, *Mucor circinelloides*, *Lichtheimia ramose*, and *Rhizopus delemar*. The first four abovementioned mold species were correlated with ≥2 key lipid metabolism-related genes, suggesting that these molds may play an important role in lipid metabolism in BQ. In addition, the first three abovementioned mold species plus *Cyberlindnera fabianii* were related to carbohydrate metabolism.

**Table 3:** Core differential microbes related to lipid metabolism in BQ.

KO	Name	Core microbes
K11533	Fatty acid synthase (Bacteria)	*Corynebacterium variabile* *Corynebacterium nuruki*
K00864	Glycerol kinase	*Lichtheimia ramosa* *Lactobacillus helveticus* *Pediococcus acidilactici*
K11262	Acetyl CoA carboxylase	*Rhizopus stolonifer* *Rhizopus delemar* *Ascoidea rubescens*
K00667	Fatty acid synthase (Fungus)	*Rhizopus stolonifer* *Mucor lusitanicus* *Lachancea kluyveri*
K00232	Acyl coenzyme A oxidase	*Rhizopus delemar* *Ascoidea rubescens* *Mucor ambiguous* *Mucor lusitanicus* *Cyberlindnera fabianii* *Mucor circinelloides*
K00249	Acyl coenzyme A dehydrogenase	Not found

The *Mucor lusitanicus* and *Rhizopus delemar* were differential core microbes related to all three metabolic pathways. *Mucor is* dominant fungi and known to be saccharification and esterase production in *Xiaoqu Baijiu* (Xiong et al., 2014; Jin et al., 2017). *Rhizopus is* dominant fungi and known to be instrumental in the aroma enhancement in *Daqu Baijiu* and *Xiaoqu Baijiu* (Hu Y. et al., 2021; Tu et al., 2022). Thus, they may be the most important microbes in BQ. The *Saccharomyces cerevisiae, Cyberlindnera fabianii, Kluyveromyces marxianus, Lactobacillus fermentum, Mucor ambiguous, Rhizopus microspores, Rhizopus azygosporus, Mucor circinelloides,* and *Ascoidea rubescens* were significantly positively correlated with two of these three metabolic pathways. *Saccharomyces cerevisiae* and *Lactobacillus* was dominant microbes in *Daqu Baijiu* and *Xiaoqu Baijiu* (Jin et al., 2017; Zhu et al., 2024). *Cyberlindnera fabianii* and *Kluyveromyces marxianus* was non-conventional yeast and has greater ester synthesis ability than *Saccharomyces cerevisiae* (Van Rijswijck et al., 2017; Karim et al., 2020). Therefore, they may be also important microbes in BQ. At present, there is no relevant report on *Ascoidea rubescens* in Chinese *Baijiu.* To be verified through future experiments.

## 4 Conclusion

In this study, the differences in microbial communities and core metabolism genes between traditional and mechanical starters of Ch*i*-flavor *Baijiu* from a Cantonese *Baijiu* factory were investigated using metagenomic technology. The core differential microbes were identified based on a correlation analysis using the NR database. Several core microbes related to carbohydrate, amino acid, and lipid metabolism were differentially enriched in the traditional starter. *Mucor lusitanicus* and *Rhizopus delemar* were significantly positively correlated with all three key metabolic pathways, i.e., carbohydrate, lipid, and amino acid metabolism. *Saccharomyces cerevisiae, Cyberlindnera fabianii, Kluyveromyces marxianus, Lactobacillus fermentum, Mucor ambiguous, Rhizopus microspores, Rhizopus azygosporus, Mucor circinelloides,* and *Ascoidea rubescens* were significantly positively correlated with two of these three metabolic pathways. The results of this study provide a basis for understanding the differences in core microbes between traditional and Round-Koji-mechanical starters, and they also provide guidance for improving Round-Koji-mechanical starter. For example, to increase the level of core microbes in the Round-Koji mechanical starter by optimizing the making conditions of starter. Further core microbes screening and identification will be conducted to gain deeper insights into the two starter types.

## Data availability statement

The datasets presented in this study can be found in online repositories. The names of the repository/repositories and accession number(s) can be found in the article/Supplementary material.

## Author contributions

JL: Conceptualization, Data curation, Formal analysis, Funding acquisition, Investigation, Methodology, Project administration, Resources, Software, Supervision, Validation, Visualization, Writing – original draft, Writing – review & editing. LD: Data curation, Formal analysis, Investigation, Methodology, Software, Writing – original draft. ZL: Investigation, Methodology, Writing – original draft. YF: Supervision, Writing – review & editing. WB: Supervision, Writing – review & editing. WZ: Supervision, Writing – review & editing. SH: Supervision, Writing – review & editing. RC: Supervision, Writing – review & editing.

## Footnotes

^1^https://www.ncbi.nlm.nih.gov/refseq/about/nonredundantproteins/

^2^http://www.genome.jp/kgee/
