# Supplementary material for: Metagenomic analysis of core differential microbes between traditional starter and Round-Koji-mechanical starter of Chi-flavor Baijiu
Source: Front Microbiol. 2024 Jun 17;15:1390899. doi: 10.3389/fmicb.2024.1390899 (PMC11215056; doi:10.3389/fmicb.2024.1390899)
Supplement: Supplementary file 1 [file Data_Sheet_1.docx]

Metagenomic Analysis of Core Differential Microbes between Traditional Starter and Round-Koji-Mechanical Starter of *Chi*-Flavor *Baijiu*

Supplementary Material

**Table S1. The information of sequencing data**

| Information | BQ_1 | BQ_2 | BQ_3 | SQ_1 | SQ_2 | SQ_3 |
| --- | --- | --- | --- | --- | --- | --- |
| Raw reads | 43186778 | 43766928 | 43326132 | 46938118 | 43243234 | 43398826 |
| Raw base (bp) | 6521203478 | 6608806128 | 6542245932 | 7087655818 | 6529728334 | 6553222726 |
| Clean reads | 42332134 | 42931284 | 42517256 | 45980864 | 42238724 | 42611378 |
| Clean base | 6379563066 | 6465188608 | 6404164281 | 6901522958 | 6366546273 | 6417405391 |
| Percent in raw reads (%) | 98.02 | 98.09 | 98.13 | 97.96 | 97.67 | 98.18 |
| Percent in raw bases (%) | 97.82 | 97.82 | 97.88 | 97.37 | 97.50 | 97.9 |
| Contigs | 154305 | 107446 | 141980 | 203600 | 157882 | 176688 |
| Contigs bases (bp)  N50 (bp) | 173207936  1941 | 136250947  2132 | 173381014  3052 | 213509535  1555 | 172248202  2178 | 211862574  2055 |
| N90 (bp) | 416 | 466 | 411 | 416 | 402 | 440 |
| ORFs | 235791 | 178175 | 231823 | 296564 | 235399 | 285110 |
| Total length (bp) | 104423733 | 81937293 | 106412067 | 125694918 | 100462755 | 122086653 |
| Average length(bp) | 442.87 | 459.87 | 459.02 | 423.84 | 426.78 | 428.21 |
| ORFs Max(bp)  ORFs min(bp) | 12903  102 | 12177  102 | 35271  102 | 16086  102 | 16086  102 | 16086  102 |


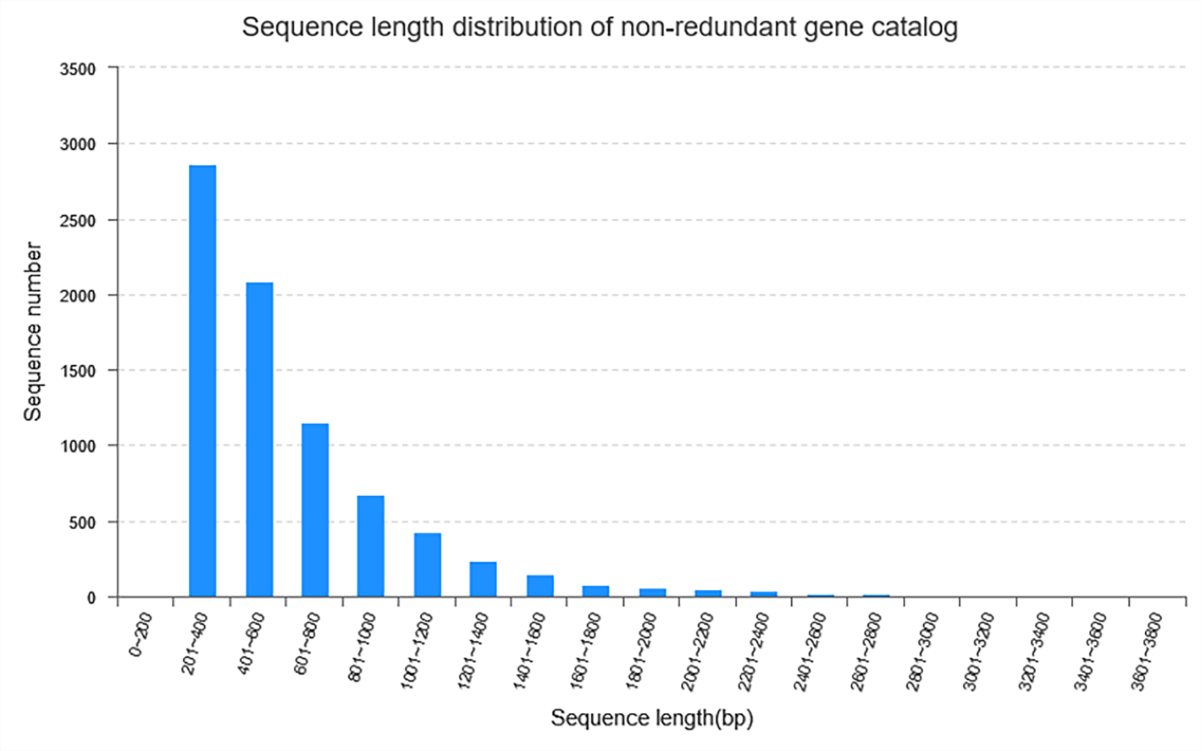


**Figure S1. Distribution of non-redundant gene lengths.**


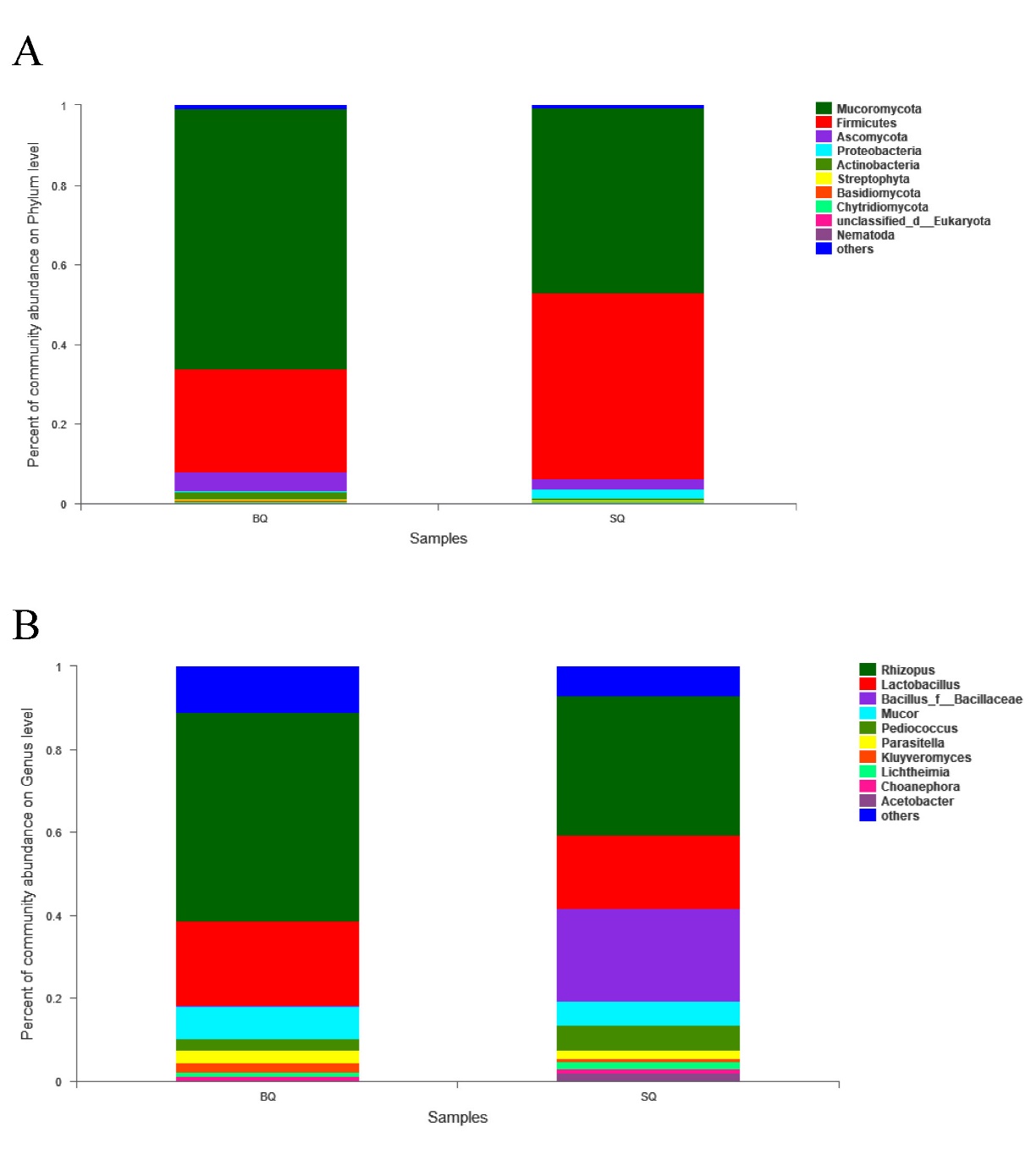


**Figure S2. The relative abundance of microbes in phylum level (A) and genus level (B) between two starters.**


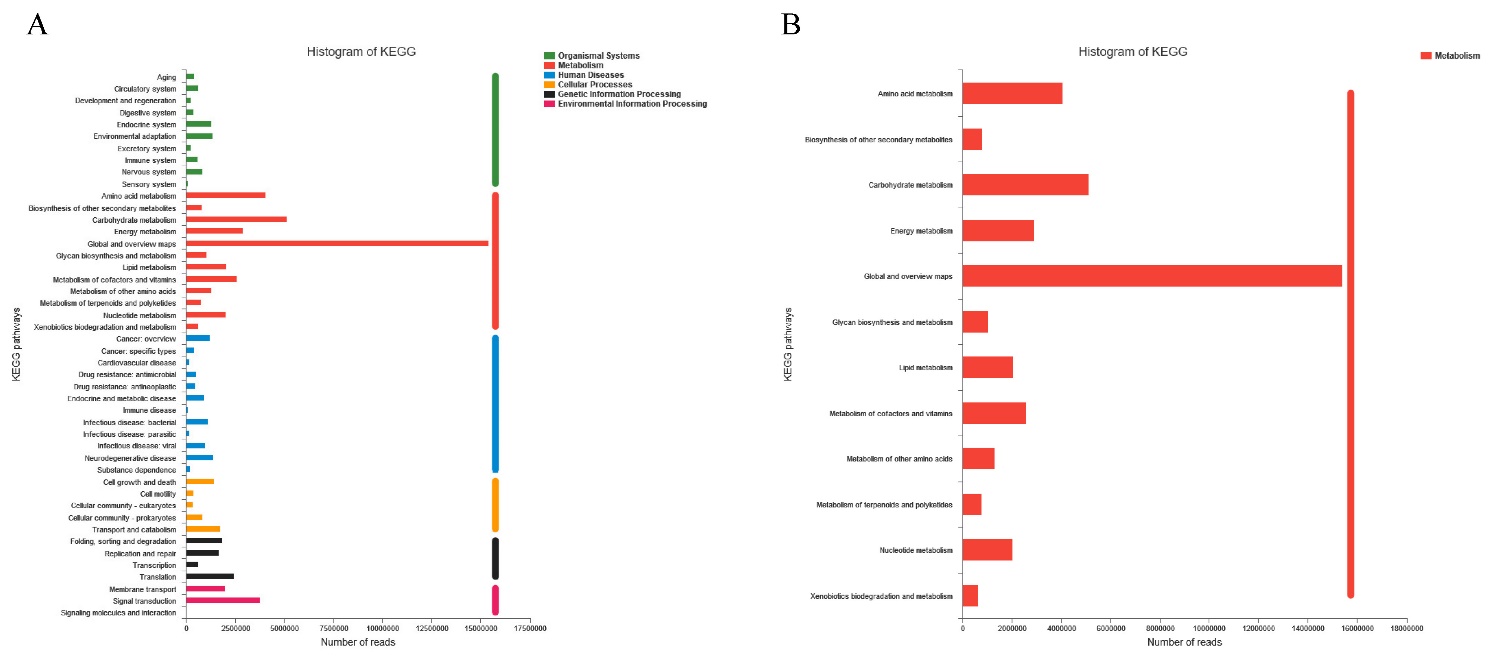


**Figure S3. Annotated pathway by KEGG at level 1 (A) and level 2 (B).**


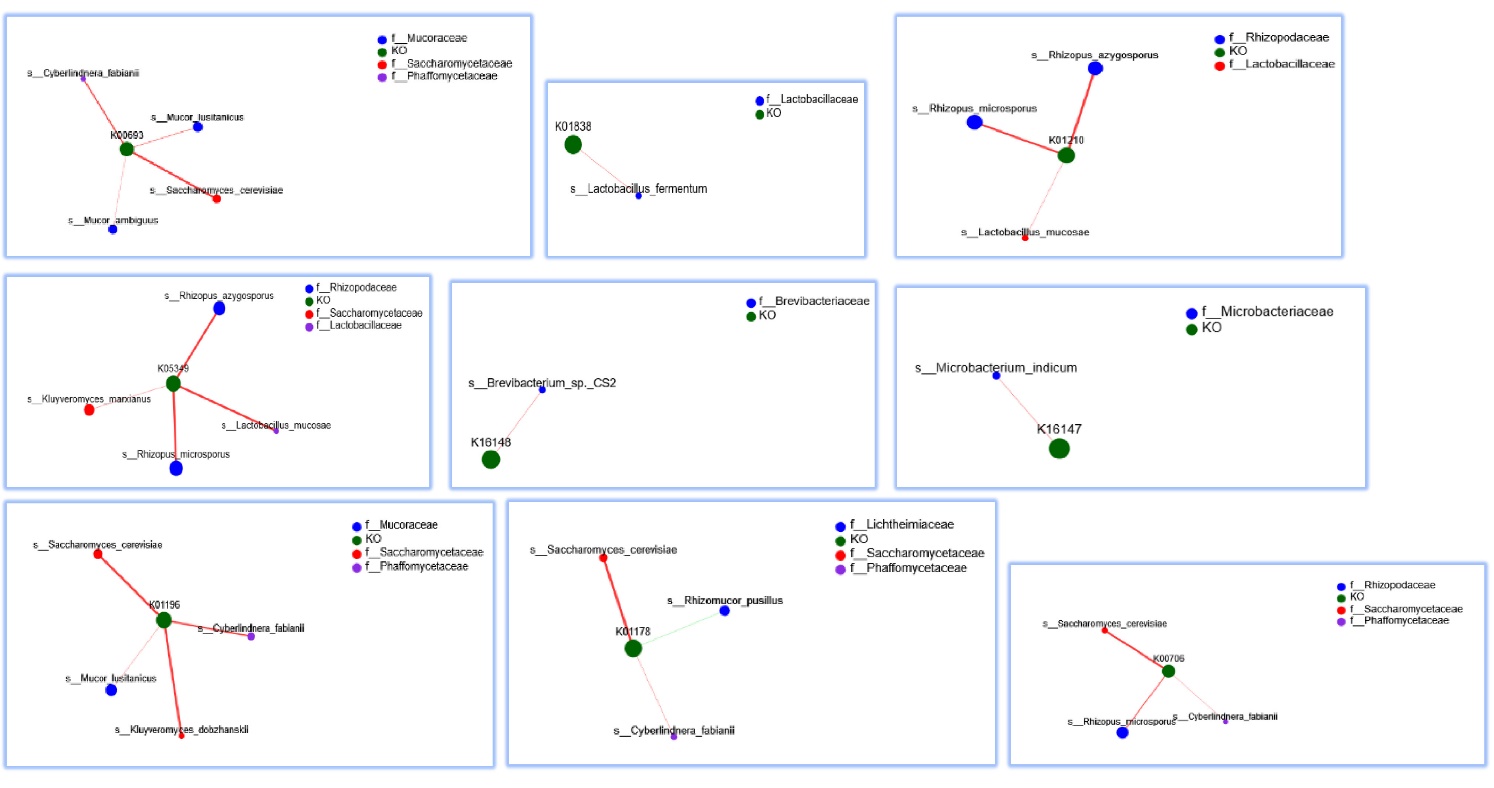


**Figure S4. Correlation analysis of carbohydrate metabolism by the NR database.**


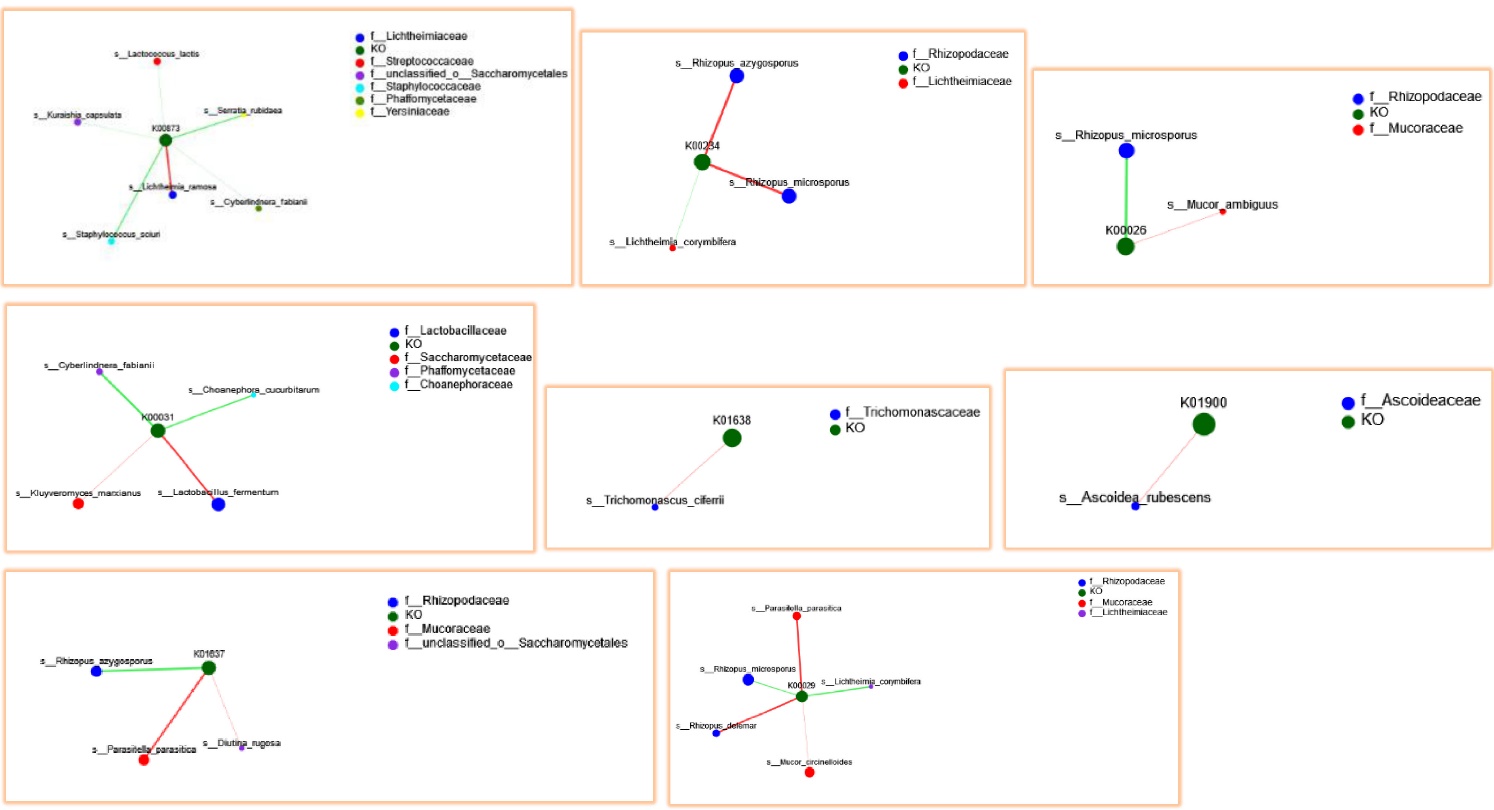


**Figure S5. Correlation analysis of amino acid metabolism by the NR database.**


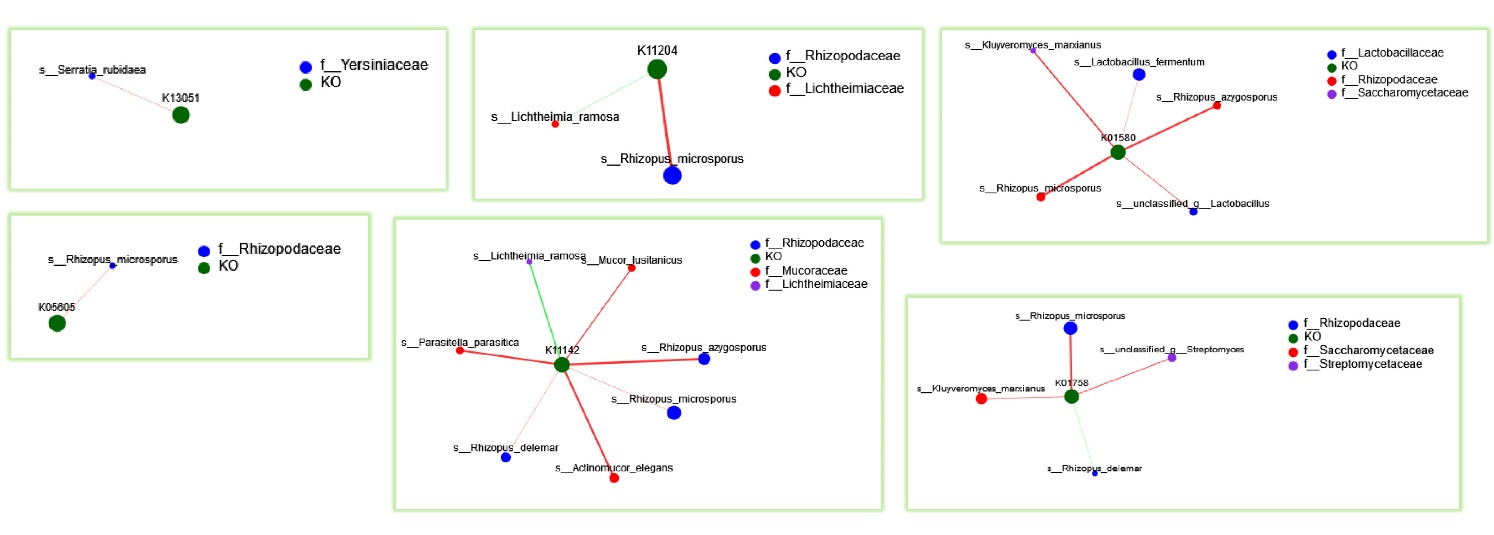


**Figure S6. Correlation analysis of lipid metabolism by the NR database.**
